# Impact of PFOS Exposure on Murine Fetal Hematopoietic Stem Cells, Associated with Intrauterine Metabolic Perturbation

**DOI:** 10.1021/acs.est.5c02623

**Published:** 2025-03-13

**Authors:** Wang Ka LEE, Hin Ting WAN, Zheyu CHENG, Wing Yee CHAN, Thomas Ka Yam LAM, Keng Po LAI, Jianing WANG, Zongwei CAI, Chris Kong Chu WONG

**Affiliations:** † Croucher Institute for Environmental Sciences, Department of Biology, Hong Kong Baptist University, Hong Kong SAR; ‡ State Key Laboratory in Environmental and Biological Analysis, Hong Kong Baptist University, Hong Kong SAR; § Department of Applied Science, Hong Kong Metropolitan University, Hong Kong SAR

**Keywords:** metabolome, transcriptome, cytokine, flow cytometry, colony-forming unit assay

## Abstract

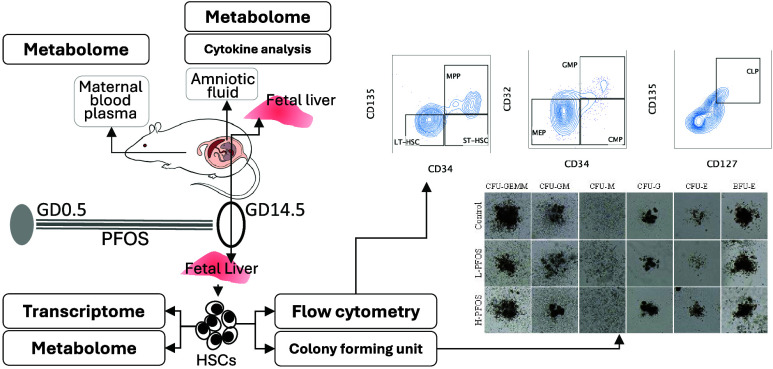

This study hypothesized that perfluorooctanesulfonate (PFOS) exposure disrupts maternal-fetal metabolism, affecting fetal liver hematopoietic stem cell (FL-HSC) development. Pregnant mice received PFOS (0.3 and 3 μg/g bw) and were sacrificed on gestation day 14.5. Metabolomic analysis of maternal plasma revealed disruptions in steroid hormone, purine, carbohydrate, and amino acid metabolism, which aligned with the enriched pathways in amniotic fluid (AF). FL analysis indicated increased purine metabolism and disrupted glucose and amino acid metabolism. FL exhibited higher levels of polyunsaturated fatty acids, glycolytic and TCA metabolites, and pro-inflammatory cytokine IL-23, crucial for hematopoiesis regulation. Transcriptomic analysis of FL-HSCs revealed disturbances in the PPAR signaling pathway, pyruvate metabolism, oxidative phosphorylation, and amino acid metabolism, correlating with FL metabolic changes. Metabolomic analysis indicated significant rises in glycerophospholipid and vitamin B6 metabolism related to HSC expansion and differentiation. Flow cytometric analysis confirmed increased HSC populations and progenitor activation for megakaryocyte, erythrocyte, and lymphocyte lineages. The CFU assay showed a significant increase in BFU-E and CFU-G, but a decrease in CFU-GM in FL-HSCs from the H-PFOS group, indicating altered differentiation potential. These findings provide for the first time insights into the effects of PFOS on maternal-fetal metabolism and fetal hematopoiesis, highlighting implications for pollution-affected immune functions.

## Abstract 2

PFOS exposure disrupted maternal-fetal metabolism and fetal hematopoiesis.

## Introduction

Exposure to environmental pollution accounted for over 16% of deaths worldwide and is the second leading cause of noncommunicable diseases.^1,2^ The disease risk associated with environmental pollution has risen by over 66% since 2000.^2^ Throughout the lifespan, the influences of environmental chemicals can have diverse effects, but *in-utero* exposure to environmental contaminants was recognized as the priority concern of chemical toxicity to fetuses. Notably, intergenerational exposure to environmental chemicals affects maternal health, disrupts placental physiology, and impairs fetal growth and developmental programming, increasing the risk of preterm health issues and disease susceptibility later in life.^3,4^ Per- and poly fluoroalkyl substances (PFASs) are significant chemical families known as “forever chemicals” that persist in public water systems, foods, air, and linger in the environment, wildlife, and humans.^5^ The chemicals exhibited a proteinophilic attraction to albumin and various fatty acid-binding proteins, leading to their extended biological half-lives and bioaccumulation in humans.^6,7^

A considerable number of studies have reported that *in-utero* exposure to PFAS is linked to developmental risk in offspring.^8−10^ The PFAS-related preterm health risk was linked to disruptions in placental function and direct chemical toxicities to fetuses, resulting in significant alternations in developmental programming.^11−13^ PFAS shortened gestation length^14^ and hindered intrauterine growth in animal and human studies.^15−17^ A meta-analysis identified maternal perfluorooctanesulfonate (PFOS) was linked to an increased risk of preterm birth.^18^ A recent metabolomic study of newborn African Americans revealed PFAS-perturbed biological pathways associated with impaired tissue neogenesis, neuroendocrine function, and redox homeostasis.^14,15^ In addition to metabolic disturbances in early life exposome,^19,20^ PFAS is known to induce inflammation and immune dysfunction, which significantly contribute to the development of chronic diseases.^21,22^

Metabolism, inflammation, and immune response are integrated with health and disease development.^23^ The proper functioning of each depends on the others.^24^ PFAS exposure has been linked to diminished antibody responses to vaccines,^25^ heightened hypersensitivity,^26^ and immune system suppression in humans.^27−29^ Experimental animal studies indicated that PFAS exposure led to immunological changes, including reductions in lymphoid organ weights, alternations in the thymus and splenic lymphocyte subpopulations, increased hypocellularity in bone marrow, and atrophy of the mandibular and mesenteric lymph nodes.^30^ The studies suggest that PFAS disturbs immune functions. However, there is no information about the potential effects of PFAS on fetal hematopoiesis. This critical process influences postnatal immune functions, occurring in fetal livers (FL) during the early stages of development. Our previous study demonstrated that PFOS negatively affected placental function in mice by significantly reducing nutrient transport and increasing corticosterone levels, which contributed to the inhibitory effects on fetal growth.^31,32^ Whole-genome bisulfite sequencing of FL from PFOS-exposed dams revealed dysregulation in DNA methylation of genes related to inflammation, glucose, and fatty acid metabolism.^32^ More importantly, the FL is a vital hematopoietic organ, offering an essential microenvironment for sustaining and differentiating hematopoietic stem cells (HSCs).^33^ Disruptions in the intrauterine environment can significantly interfere with intrinsic metabolism and inflammation, impacting the development and differentiation of FL and HSCs.

During early embryonic development, HSCs and multipotent progenitors (MPPs) play a pivotal role in FL hematopoiesis.^34^ FL-HSCs rapidly increased from gestational day (GD) 11.5 to 16.5, and differentiation occurred.^35,36^ The developmental processes of FL-HSC are susceptible to nutritional perturbations.^37^ They are compromised by *in-utero* metabolic programming^38^ and inflammation.^39^ We hypothesized that *in-utero* PFOS exposure disrupts FL metabolism, negatively affecting the HSC niche. This disruption may impair the expansion and differentiation of HSCs into various blood cell lineages within the fetal liver.

## Materials and Methods

### Animals

CD-1 mice (ICR) were housed in polypropylene cages with LabDiet, water (in glass bottles), and sterilized bedding at 23–24 °C and 12 h of light/dark. Breeding was performed, and sperm-positive smears were determined to be gestational day (GD) 0.5 the following morning. Pregnant mice were housed individually and divided into three groups (control, low-, and high-dose PFOS), with each group of 6–7 mice. Food and water were freely available to mice under standard conditions. A solution of perfluorooctanesulfonate (PFOS, 98% purity, Sigma-Aldrich) was prepared by dissolving it in dimethyl sulfoxide and mixing it with corn oil. The exposed groups were administered 0.3 or 3 μg/g body weight (bw)/day of PFOS by oral gavage from GD 0.5 to 14.5. Corn oil with 0.01% dimethyl sulfoxide (DMSO) was administered to the control group. The doses were selected based on the tolerable daily intake (TDI) of PFOS in humans and were occupationally relevant, as described in our previous study.^32^ A guideline and regulation approved by the animal ethics committee of Hong Kong Baptist University (REC/22-23/0468) were followed. At GD14.5, pregnant mice underwent cervical dislocations. Changes in body and liver weights were recorded. Male fetuses were studied as a further investigation from our previous study.^31,32^ Sexing was implemented by a visual examination of fetal gonads or PCR using fetal genomic DNA.^40^ Amniotic fluid (AF) and fetal livers (FL) were collected for metabolomics and cytokine analyses (BioLegend). FL was also collected to measure liver cellularity, FL hematopoietic stem cell (FL-HSC) populations, and clonogenicity.

### RNAseq

Harvested FL (GD14.5) were pipetted gently in phosphate-buffered saline (PBS) containing 2% fetal bovine serum (FBS) and 0.5 mM ethylenediamine tetraacetic acid (EDTA) to form a single-cell suspension, then filtered through a 70 μm cell strainer. FL-HSCs were positively selected using c-Kit^+^ MicroBeads (Miltenyi Biotec, USA). Total RNA was extracted using TRIzol solution, and the RNA quality was analyzed using the Agilent 2100 Bioanalyzer. Four biological replicates per treatment with RNA integrity Number (RIN) > 8 were used for mRNA library construction (polyA enrichment). Sequencing was conducted with NovaSeq X Plus (PE150) platform. Filtering raw reads included removing adapters, reads with *N* > 10%, and reads with Qscores of over 50% bases below 5 (Novogene). Quality-trimmed sequence reads were aligned to the mouse genome reference (*Mus musculus*, GRCm39/mm39). The read-count data were then analyzed for differential expression using the DESeq 2R package (version 1.20.0).^41^ Genes were considered differentially expressed if they had a *p*-value of ≤0.05 and a |log2(fold change)| of ≥1.

### Metabolomic Analysis

Metabolite extraction was conducted on various samples, including maternal blood plasma, AF, FL (with four biological replicates), and FL-HSCs (with three biological replicates), for each treatment in individual sample analyses. Maternal blood samples (∼0.8 mL) were collected through cardiac puncture and centrifuged for 10 min at 4 °C at 2000 rpm to collect plasma. An amniotic sac puncture was performed to collect the fluid samples (∼0.1 mL). Before analysis, both samples were kept at −80 °C. Blood plasma and AF samples were mixed with methanol at a ratio of 1:4, while c-Kit-enriched FL-HSCs (1 × 10^6^) were mixed with 1 mL of 80% methanol. The samples were vortexed and incubated at −80 °C for 30 min. FL samples were minced and extracted using 80% methanol at 1:40 (w/v), mixed with magnetic beads, and homogenized using a high-speed blender (Bullot Blondor). The samples were then centrifuged at 15,000*g* for 10 min. The supernatant was evaporated to dryness and reconstituted in 50% methanol. The reconstituted metabolites were centrifuged prior to mass spectrometry (MS) analysis. The metabolomics analyses were performed using an Ultimate 3000 UPLC system coupled with a Q-Exactive mass spectrometer (Thermo Scientific), as described in our previous studies.^32,42,43^ MS data were processed and analyzed using Progenesis QI software (Nonlinear Dynamics, Newcastle, U.K.) for peak alignment, normalization, and metabolite identification. Differential analysis was performed by calculating the fold change (FC) and applying a significance threshold based on *p*-values. Metabolites with a fold change (FC) > 1.5 and *p*-value <0.05 were considered significantly altered between the groups. Statistical significance was determined using Student’s *t* test. The KEGG (Kyoto Encyclopedia of Genes and Genomes) database was utilized for metabolic pathway enrichment analysis further to investigate the biological relevance of the altered metabolites.

### Cytokine Analysis

FL was homogenized with ice-cold PBS and protease inhibitors (Thermo Fisher), followed by centrifugation at 13,000*g* at 4 °C. The supernatant was collected and diluted with an assay buffer (LEGENDplex, BioLegend), with a 1:1 ratio for FL homogenates and 1:0.5 for AF. Cytokines and chemokines were measured using the Mouse Inflammation Panel (BioLegend) according to the manufacturer’s instructions. Samples (*n* = 4) were analyzed using the FACSymphony A1 Cell Analyzer (BD Biosciences), and the data were processed using the LEGENDplex Data Analysis Software Suite.

### Flow Cytometric Analysis of FL-HSCs

Harvested FL (GD14.5) (*n* = 5) were pipetted gently in PBS containing 2% FBS and 0.5 mM EDTA to form a single-cell suspension. The cells were filtered through a 70 μm cell strainer. Multiparameter flow Cytometry, Part Analysis (BD Biosciences) was implemented using cell surface markers (Table S1) to identify the (i) long-term HSC (LT-HSC: Lin^–^, Sca1^+^, cKit^+^, CD34^–^, Flt3^–^) and (ii) short-term HSC (ST-HSC: Lin^–^, Sca1^+^, cKit^+^, CD34^+^, Flt3^–^) the quiescent populations that can self-renew and differentiate into various blood cell lineages. (iii) The multipotent progenitors (MPP: Lin^–^, Sca1^+^, cKit^+^, CD34^+^, Flt3^+^), (iv) the common lymphoid progenitor (CLP: Lin^–^, Sca1^lo^, cKit^lo^, Flt3^+^, CD127^+^); (v) common myeloid progenitor (CMP: Lin^–^, Sca1^–^, cKit^+^, CD34^+^, CD16/32^–^); (v) megakaryocyte-erythrocyte progenitor (MEP: Lin^–^, Sca1^–^, cKit^+^, CD34^–^, CD16/32^–^); (vi) granulocyte-macrophage progenitor (GMP: Lin^–^, Sca1^–^, cKit^+^, CD34^+^, CD16/32^+^). Background staining was performed with isotype-control antibodies. The flow cytometry data were analyzed using FlowJo v10.8 Software (BD Life Sciences) with the described gating strategy.^44,45^Supplementary Figure S1 shows the overall gating strategy. The changes in the percentages of the individual cell types were quantified and compared among the control and PFOS-exposed groups.

### Clonal Heterogeneity in FL-HSCs

This study aimed to determine the effects of PFOS exposure on the clonogenic potential and clonal heterogeneity of FL-HSCs. The determination of colony formation units for BFU-E (burst forming unit-erythroid), CFU-E (colony forming unit-erythroid), CFU-G (colony forming unit-granulocyte), CFU-M (colony forming unit-macrophage), CFU-GM (colony forming unit-granulocyte-macrophage), and CFU-GEMM (granulocyte-erythrocyte-megakaryocyte-macrophage) were performed in mouse methylcellulose complete media (R&D). FL-HSCs were positively selected using cKit MicroBeads (Miltenyi Biotec, USA). On each 35 mm^2^ plate, 1 × 10^4^ selected cells were aliquoted per plate containing 1.1 mL medium. The cells were incubated at 37 °C humidified incubator for 7 days. Technical duplicates were conducted, and there were four biological replicates. Cell colonies were identified and counted using light microscopy, following the manufacturer’s protocol from R&D Systems. Briefly, colonies containing erythroid progenitors appeared red. BFU-E colonies were large and dense, while CFU-E colonies comprised small clusters. CFU-M produced large, round, colorless macrophages that were homogeneous in appearance. CFU-G generated colorless granulocytes that were smaller than macrophages. CFU-GM produced a heterogeneous population of both macrophages and granulocytes. CFU-GEMM cells were found in a single colony and exhibit both reddish (erythroid) and colorless (granulocytes, macrophages, and megakaryocytes) characteristics. CFU fold changes were quantified among the control and the PFOS-exposed groups.

### Statistical Analysis

The data was analyzed using a statistical mean and standard deviation. Statistical analyses were performed using GraphPad Prism version 8.0. Data were evaluated using Student’s *t* tests. A *p*-value threshold of less than 0.05 was considered statistically significant for all analyses.

## Results and Discussion

**Figure 1:**
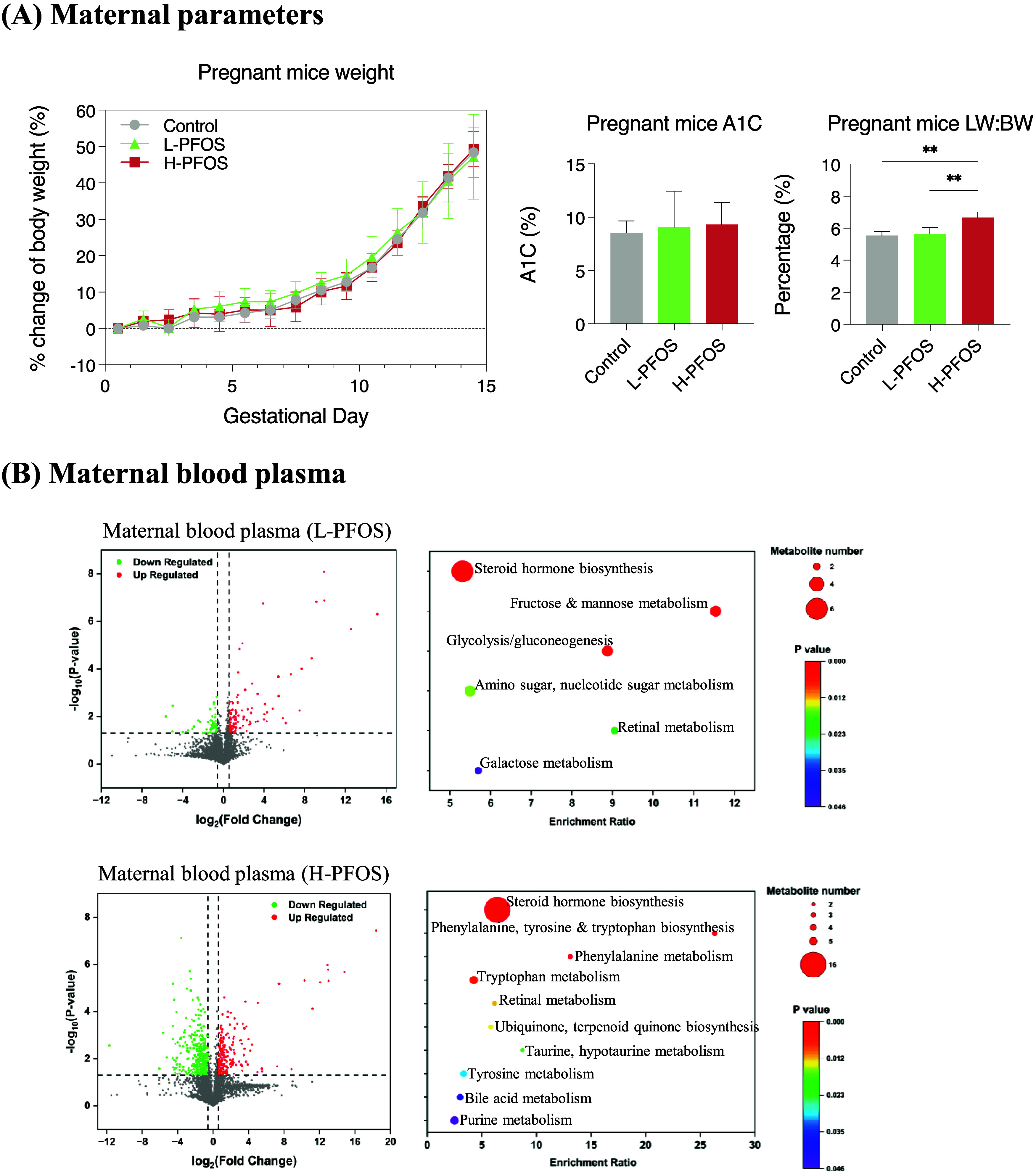
Effects of *in-utero* PFOS exposure on maternal mice’s body weight, liver weight, and blood metabolome at gestational day 14.5. (A) The growth curves of the control and PFOS-exposed groups, the levels of glycated hemoglobin (A1C), and the percentage ratio of liver weight (LW) to body weight (BW) (*n* = 6). (B) The scatter plots of volcano analysis for dysregulated metabolites (the *x*-axis represents the fold change in metabolites between the control and PFOS samples, while the *y*-axis indicates the statistical significance of the differences. Red dots denote upregulated metabolites, whereas green dots signify downregulated metabolites) and KEGG enrichment (the abscissa in the graph is the ratio of the number of differential metabolites on the KEGG pathway to the total number of differential metabolites, and the ordinate is the KEGG pathway) in maternal blood plasma (*n* = 4). KEGG enrichment analysis reveals significantly altered pathways in the L-PFOS and H-PFOS groups. Data were presented as the mean ± standard deviation (SD). **P (control vs L-HPOS & H-PFOS) < 0.01.

In this study, *in-utero* PFOS exposure showed no significant changes in maternal body weight or glycated hemoglobin (HbA1c) levels at GD14.5. However, there was a significant increase in the ratio of liver weight to body weight in the H-PFOS group (Figure 1A). The observation supports our earlier study that PFOS exposure impaired β-oxidation and reduced liver lipid export, leading to hepatic lipid accumulation.^43^ To investigate metabolic perturbations further, maternal blood plasma samples were analyzed for metabolomic profiles (Table S2). The analysis revealed disturbances in steroid hormones and metabolism of purines, carbohydrate, amino acids, and their derivatives (Figure 1B). In hindsight, mitochondrial P450-mediated steroidogenesis has been reported to be influenced by PFAS exposure.^46−48^ Studies have often linked PFOS-induced purine metabolism to increased oxidative stress.^49,50^ Higher serum levels of PFAS in adult humans were associated with increased uric acid levels,^51^ a natural byproduct of purine metabolism. Since purine metabolism is a crucial biochemical process that regulates nucleic acid synthesis and cellular energy production, its dysregulation is linked to oxidative stress and metabolic diseases.^52,53^ The altered metabolism of carbohydrates and amino acids represents common metabolic signatures of PFAS in maternal serum metabolomics.^15,54,55^ Collectively, our data illustrated that PFOS disrupted the maternal metabolic environment.

**Figure 2:**
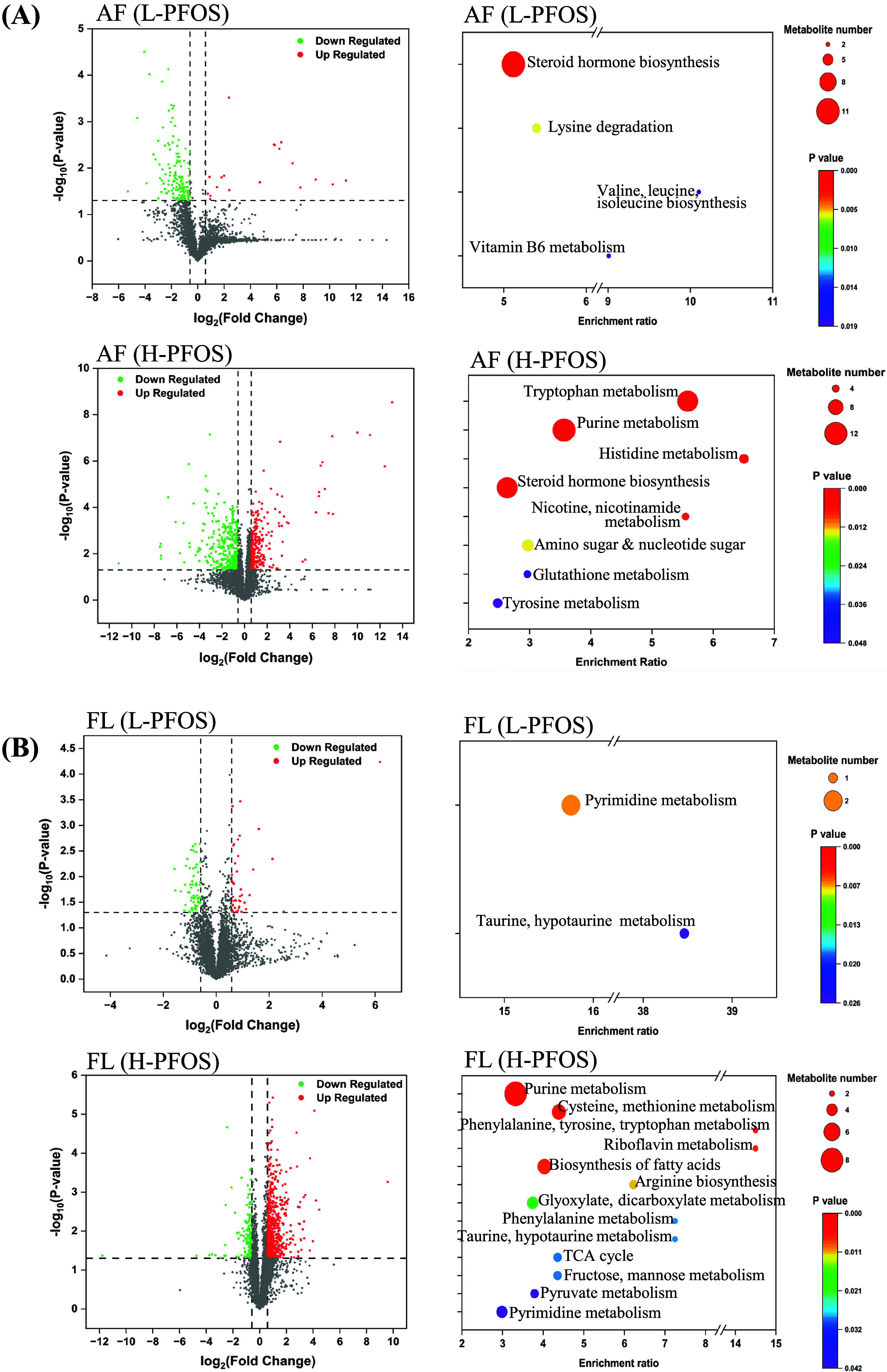

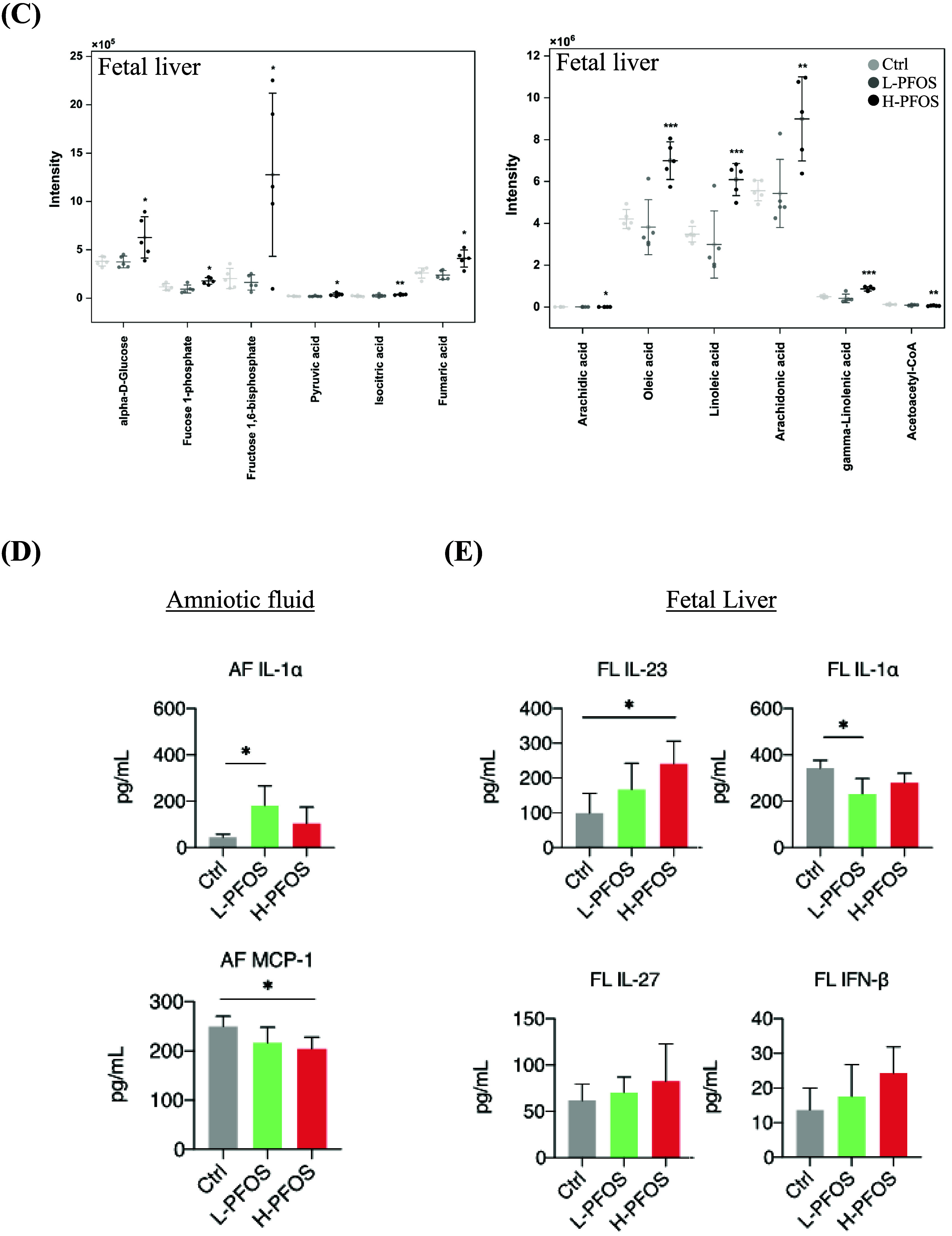
Metabolome and cytokine levels in the amniotic fluid and fetal livers at gestation day 14.5. (A) Amniotic fluid (AF)—the scatter plots of volcano analysis for dysregulated metabolites and KEGG enrichment (*n* = 4). KEGG enrichment analysis reveals significantly altered pathways in the L-PFOS and H-PFOS groups. (B) Fetal liver (FL)—the scatter plots of volcano analysis and KEGG enrichment (*n* = 4). (C) Significant changes in the metabolites of glucose and fatty acid metabolism were observed in the L-PFOS and H-PFOS groups. The levels of cytokines in (D) AF and (E) FL. Data were presented as the mean ± SD. *P (control vs L-PFOS & H-PFOS) < 0.05; **P < 0.01 and ***P < 0.001.

Mounting evidence suggests maternal metabolism shapes fetal metabolism during development.^56^ Our previous studies showed that perturbed maternal metabolism negatively affected fetal growth^31,57^ and FL metabolism.^32^ In this study, amniotic fluids (AF) and FL were collected to reveal changes in metabolic and inflammatory parameters, reflecting the intrauterine environment. AF is derived from maternal plasma via the embryonic membrane and reflects fetal metabolism.^58,59^Supplementary Figure S2A shows the dose-dependent increases in PFOS concentrations in AF and FL. Figure 2A presents the scatter plots of volcano analysis for dysregulated metabolites and KEGG enrichment in AF. The specific pathways that were down- and up-regulated are shown in Supplementary Figure S2B and Table S3. In AF, the dysregulated metabolism of steroid hormones, amino acids (tryptophan, tyrosine, amino acid sugars, nucleotide sugars), and purines was consistently observed in maternal blood samples (Figure 1B). The metabolic pathway for steroid hormone synthesis was the most significant enrichment in analyses of both maternal and AF pathways. Supplementary Figure S2C showed significantly reduced levels of most steroid hormones, including corticosteroids, dihydroxy-pregnenolone, and androgens. Corticosteroids are essential for fetal organ maturation,^60^ while sex steroids are essential for offspring’s brain and reproductive health.^61,62^ These observations could provide additional insight into the reported effects of prenatal PFOS exposure on fetal growth,^14^ neurodevelopment,^63^ and reproductive health.^64^ Additionally, tryptophan and purine metabolism were the other two pathways significantly enriched in maternal blood and AF analyses (Figure 2A). The disruption of tryptophan metabolism has been identified as a significant metabolomic signature linked to PFAS-potentiated preterm birth.^14^ An experimental study also demonstrated the binding interaction of PFOS with tryptophan and DNA, suggesting the potential molecular toxicity of PFAS.^65^ In retrospect, tryptophan metabolism is linked to the production of tryptamine, kynurenine, serotonin, and indole—all of which play roles in immunological processes during pregnancy.^66,67^ Changes in steroid hormone and purine metabolism in maternal blood may be reflected in the enriched pathways in AF.

Consistently, purine metabolism was upregulated in the FL metabolomic analysis, alongside the amino acids (tryptophan and tyrosine), their derivatives (taurine and hypotaurine), fatty acids, carbohydrates, and TCA cycle (Figure 2B), which aligned with the maternal blood and AF data. The pathways that were down-regulated and up-regulated in FL are displayed in Supplementary Figure S2D and Table S4. Notably, there was a significant upregulation of key glycolytic intermediates, including glucose, fructose-1 phosphate, fructose-1,6-bisphosphate, and pyruvic acid, as well as TCA cycle metabolites, such as fumaric acid and isocitric acid (Figure 2C, left panel). The data suggest heightened glycolysis and mitochondrial metabolism to support FL metabolism^68,69^ and HSC expansion.^70^ Conversely, pathways like glyoxylate/dicarboxylate metabolism, arginine biosynthesis, and taurine metabolism were downregulated (Supplementary Figure S2D). The pathways were linked to liver metabolic functions in humans affected by PFAS,^71^ potentially affecting normal hematopoiesis. In HSC transplantation therapies, the development of acute myeloid leukemia is linked to dysfunctions in cellular metabolic pathways, with glyoxylate and dicarboxylate metabolism identified as one of the foremost canonical pathways.^72^ Extracellular arginine is crucial for maintaining HSCs,^73^ while taurine has antioxidant properties, and its conjugate protects HSCs from unfolded protein stress in FL.^74^ Since PFOS mainly impacts lipid metabolism and fatty acid signaling, we further analyzed the pathways related to fatty acid metabolites. In the H-PFOS group, we observed significant increases in polyunsaturated fatty acids (PUFA), oleic acid, linoleic acid, linolenic acid, and arachidonic acid (Figure 2C, right panel). These increases have been consistently reported alongside PFOS exposure in animal^42,75^ and human studies.^14,76^ PUFAs activate PPAR signaling,^77^ which regulates hematopoiesis.^78−80^ Collectively, the AF and FL metabolomic analysis suggests that PFOS exposure disrupted the intrauterine metabolic environment. In the cytokine analysis, AF showed increased IL-1α in the L-PFOS group, while MCP-1 decreased in the H-PFOS group (Figure 2D), which aligns with our earlier findings in mouse placentas exposed to PFOS *in-utero*.^32^ Cytokine analysis in FL showed decreased IL-1α in the L-PFOS group and increased IL-23 in the H-PFOS group (Figure 2E). Other measured cytokines in AL and FL showed no significant changes (Figure S3A,B). IL-1α and IL-23 are pro-inflammatory cytokines that regulate HSC function.^81^ Given that HSCs develop in FL, the high levels of IL-23 likely have a considerable effect on HSC development. In hindsight, HSCs are a primary target of the IL-23-driven inflammatory pathway that promotes granulocyte production lineage.^82^

**Figure 3:**
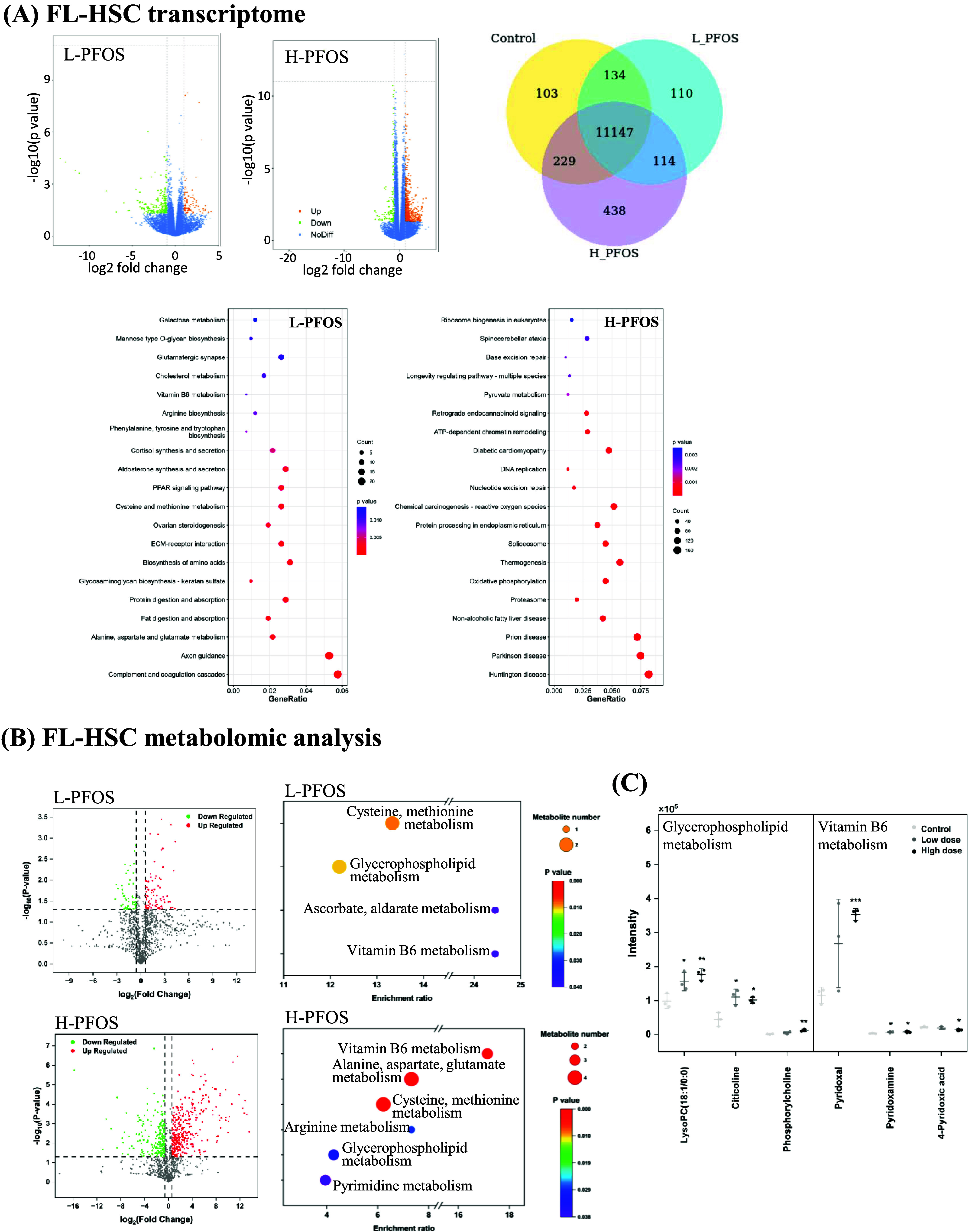
Transcriptome and metabolome of FL-HSCs at gestational day 14.5. (A) Upper left: volcano plots of differentially expressed genes (*n* = 4). The *x*-axis represents the fold change in gene expression between the control and PFOS-exposed groups, while the *y*-axis indicates the statistical significance of the differences. Red dots denote upregulated genes, and green dots denote downregulated genes. Upper right: the coexpression Venn diagram illustrates the number of genes uniquely expressed within individual groups, while the overlapping areas indicate the number of genes coexpressed in two or more groups. The lower panel presents the KEGG enrichment. The analysis mainly focuses on metabolic and cellular signaling pathways, offering detailed maps of biochemical reactions and gene interactions. The abscissa in the graph represents the ratio of the number of differential genes in the KEGG pathway to the total number of differential genes, while the ordinate indicates the KEGG pathways. The 20 most significant KEGG pathways were displayed. The point size indicates the number of genes annotated to a specific KEGG pathway, while the color gradient from red to purple reflects the significance level of enrichment. (B) The metabolome volcano plot and KEGG enrichment analysis reveal the differing profiles of metabolites and pathways between the control and PFOS groups (left) (*n* = 3). (C) The graph shows the significant changes in the associated metabolites of glycerophospholipids and vitamin B6 metabolism. Data were presented as the mean ± SD. *P (control vs L-PFOS & H-PFOS) < 0.05; **P < 0.01 and ***P < 0.001.

The FL metabolic and inflammatory environment influences hematopoiesis, regulating HSC metabolism for maintenance and differentiation.^68^ To address this, FL-HSCs were enriched using a c-Kit^+^ magnetic column (Figure S4A), followed by RNAseq (Table S5) and metabolomic analyses (Table S6) to provide a comprehensive approach for investigating how the PFOS-induced changes influence the metabolic networks and biological pathways related to HSC function. Supplementary Figure S4B presents the differential expression gene clustering heatmap of cKit^+^-enriched FL-HSCs from the control and *in-utero* PFOS exposure at GD14.5. Figure 3A (upper panel) shows the volcano plot analysis, while the Venn diagram reveals dysregulated genes, indicating a high number of uniquely expressed genes in the H-PFOS (438) group compared to the L-PFOS (110) and control (103) groups. Data showed that H-PFOS exposure significantly affected FL-HSC gene expression. The KEGG analysis indicated significant disturbances in the PPAR signaling pathway, pyruvate metabolism, oxidative phosphorylation, and amino acid metabolism (Figure 3A, lower panel), aligning with the metabolic changes in FL (Figure 2B). PFOS is a selective modulator of PPAR receptors. The disruption of PPAR pathways affects fatty acid oxidation^83^ and mitochondrial metabolism,^84^ both of which are vital for maintaining and expanding HSCs. Additionally, significantly altered pathways relate to cell growth, differentiation, and immune functions, covering chromatin remodeling, DNA replication, spliceosome activity, ribosome biogenesis, endoplasmic reticulum function, proteostasis, ECM-receptor interactions, and the complement cascade (Figure 3A, lower panel). Reactome analysis revealed additional affected pathways, such as extracellular matrix organization, mitochondrial function, and RNA/protein metabolism (Figure S4C), which are known to influence stem cell development behavior.^85−87^

FL-HSC’s metabolome identified significantly altered pathways (Figure 3B). Two commonly upregulated pathways were identified in the L-PFOS and H-PFOS groups (Figure S5), including the metabolism of glycerophospholipid (GPL) and vitamin B6. GPLs are key structural lipids in various cellular processes, including cell division, vesicle trafficking, and signal transduction.^88^ GLP metabolic pathway was enriched in newborn dried blood spot metabolome of PFAS-exposed African Americans.^14^ Elevated GPL levels were associated with aberrant myeloid expansion via activation of Toll-like receptor-4 signaling in a human study,^89^ affecting normal hematopoiesis. In this study, the GPL-related metabolites (lysoPC, citicoline, and phosphorylcholine) were significantly upregulated in the H-PFOS groups (Figure 3C) and were involved in the regulation of hematopoiesis.^90,91^ Additionally, a recent study identified that vitamin B6 metabolism was associated with prenatal PFOS exposure in the American maternal-child cohort.^76^ The higher levels of vitamin B6 are crucial for cellular metabolism and maintenance in embryonic stem cells^92^ and were shown to lessen inflammatory effects on HSC proliferation and lineage fate.^93^ In this study, significant increases in pyridoxine, pyridoxal, and pyridoxamine (the different forms of vitamin B6) were observed in the H-PFOS group (Figure 3C). In addition to functioning as coenzymes and antioxidants, vitamin B6 metabolites also serve as iron chelators,^94^ essential for the proper expansion and differentiation of HSCs.^95^ Collectively, the altered pathways and metabolites were directly associated with the regulation of HSC maintenance and differentiation.^68,96^ Further experiments were prompted to investigate the functional phenotypes of FL-HSCs.

**Figure 4:**
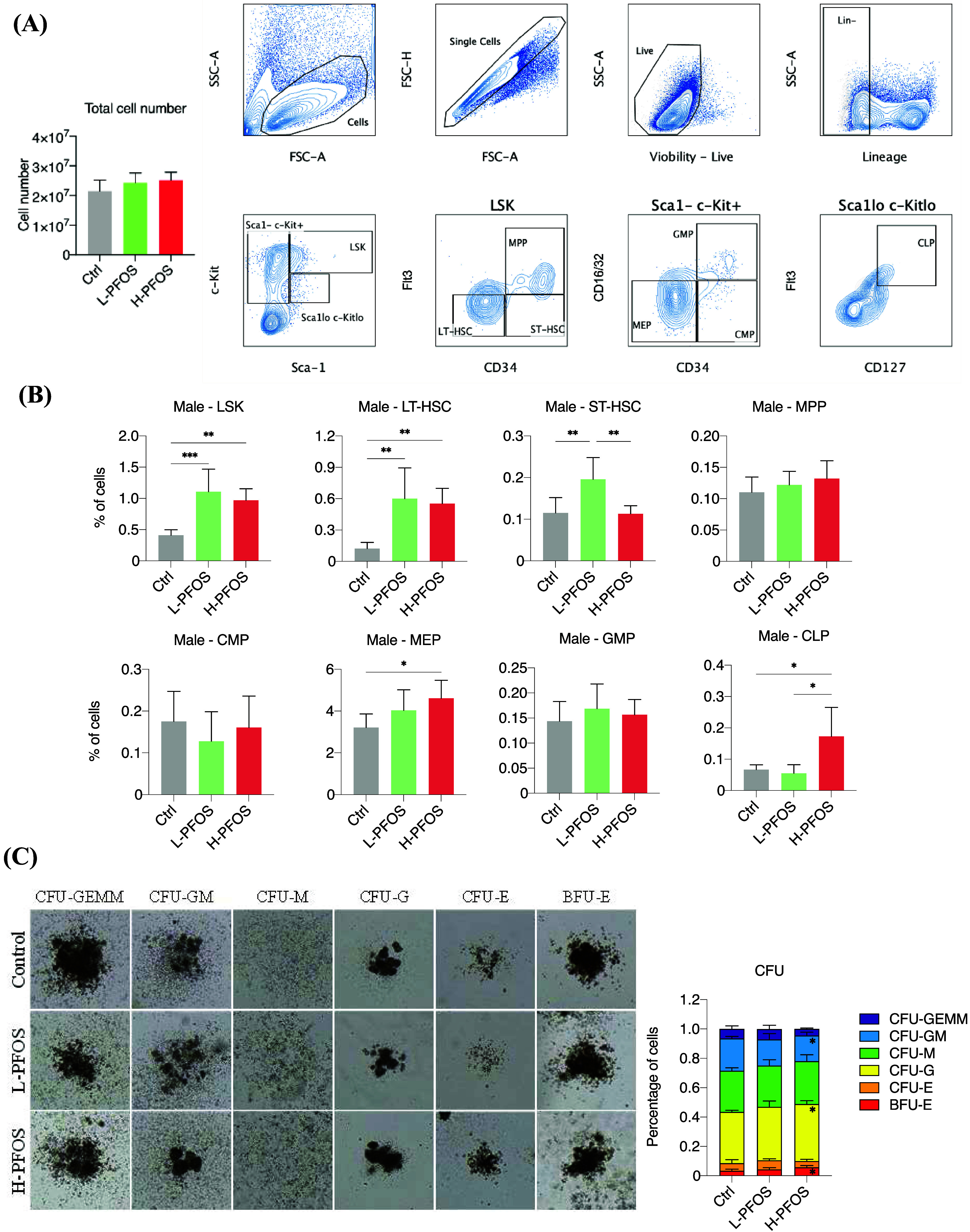
Flow cytometric and CFU analysis of FL-HSCs at gestational day 14.5. (A) Left: the total number of fetal liver cells in the control and PFOS groups (*n* = 6). Right: A representative flow diagram shows the gating of fetal liver cells, lin-, LSK, Sca^–^cKit^+^, and Sca^lo^cKit^lo^ for the identification of “LT-HSC, ST-HSC, MPP,” “MEP, CMP, GMP,” and “CLP” populations, respectively. (B) Quantitative differences in LSK, LT-HSC, ST-HSC, and other lineages (*n* = 5). (C) The colony-forming unit assay reveals changes in the formation of different lineages using FL-HSCs isolated from control and PFOS-exposed fetuses at GD14.5 (*n* = 4). Left: light microscopy images of representative colonies. Right: the tabulated counts of colonies in the control and PFOS groups. Data were presented as the mean ± SD. *P (control vs L-PFOS & H-PFOS) < 0.05. **P < 0.01.

To investigate how *in-utero* PFOS exposure impacts the maintenance and differentiation of fetal liver HSCs at GD14.5, we conducted multiparameter flow cytometric analyses to profile freshly isolated HSCs and assess the heterogeneity of the cell populations. In Figure 4A, we presented the total fetal liver cell number and a representative flow cytometry gating of fetal liver cells, where viable cells were identified and gated. HSCs were distinguished from non-HSCs based on Lin^–^, Sca-1^+^, and c-Kit^+^ staining. Long-term (LT)-HSCs, short-term (ST)-HSCs, and multipotent progenitors (MPP) were further identified using additional markers (CD34, Flt3). Common myeloid progenitor (CMP), granulocyte/monocyte progenitor (GMP), and megakaryocyte/erythroid progenitor (MEP) populations were characterized with specific antibodies (CD16/32 & CD34), while common lymphoid progenitors (CLP) were identified using CD127 and Flt3. Figure 4B summarizes the flow cytometric data, indicating that *in-utero* PFOS exposure altered hematopoietic development, potentially increasing the LSK cell population and affecting LT- and ST-HSCs. There was no significant difference in ST-HSCs between the control and H-PFOS. However, L-PFOS treatment resulted in a significant increase in ST-HSCs. Based on our data, this was likely attributable to an increase in HSC differentiation at H-PFOS, as evidenced by a significant increase in the MEP and CLP. While flow cytometry characterized various HSC populations based on cell surface markers, it did not directly assess their functional capabilities. Therefore, we conducted CFU assays to measure the differentiation potential of these cells into various blood cell lineages, as visualized through colony morphology (Figure 4C, left panel). The tabulated data on the percentage changes of six colony types: BFU-E (erythroid), CFU-E (erythroid), CFU-G (granulocyte), CFU-M (macrophage), CFU-GM (granulocyte/macrophage), and CFU-GEMM (granulocyte/erythrocyte/macrophage/megakaryocyte) was shown (Figure 4C, the right panel). Our results revealed a significant increase in BFU-E and CFU-G, but a significant decrease in CFU-GM in FL-HSCs from the H-PFOS group.

The PFAS elicited preterm health risks and reduced the length of gestation.^14,15^ Linking our findings with previous epidemiological studies, it is noteworthy that preterm cord blood contains significantly higher concentrations of HSCs and primitive progenitors compared to term cord blood,^97−99^ indicating an enhanced clonogenic capacity of HSCs in preterm neonates. Furthermore, our flow data revealed a significant increase in MEP percentage in the H-PFOS group, which is critical for generating BFU-E in erythropoiesis. This finding aligns with our CFU assay data, demonstrating a significant increase in BFU-E, suggesting heightened proliferation and differentiation of erythroid progenitors. Interestingly, similar changes were reported in cord blood samples from preterm neonates, which exhibited higher HSC levels and elevated hemoglobin.^100^ The growth of BFU-E and CFU-G from preterm cord blood was higher than that from full-term infants.^101^ Considering the effects of PFOS on intrauterine restriction, the notable increase in the proliferation and differentiation of erythroid progenitors may represent a compensatory response to PFOS exposure *in-utero*. In this study, the increase in CFU-G and the decrease in CFU-GM suggested that hematopoiesis prioritized granulocyte production over macrophage lineage following PFOS exposure. This may reflect an *in situ* response of HSCs to elevated FL IL-23 levels (Figure 2E), activating the inflammatory pathway and increasing the demand for granulocytes, key immune components involved in inflammation.^82^ Notably, PFOS can activate danger-associated molecular patterns by causing cellular injury and releasing nonoxidized mitochondrial DNA. This release stimulated pattern recognition receptors, particularly AIM2, leading to “sterile inflammation” and the release of inflammatory cytokines.^102^ These processes generally promote the proliferation and differentiation of HSCs^103^ and the expansion of lymphoid-biased progenitors in the fetus.^39^ In the H-PFOS group, our data indicated a significant increase in the population of CLP. This increase was associated with the fetal development of immune tolerance, which may elevate the risk of developing hematological malignancies and impact the ability to respond to infections later in life.^104,105^ Immune dysregulation *in-utero* is a hypothesized mechanism for the development of leukemia.^106^ Our findings may provide insight into the epidemiological links between childhood leukemia^107^ and deficient immune response^108,109^ associated with PFAS exposure in developing fetuses. Collectively, PFOS-affected hematopoiesis may negatively impact the development of the immune system, potentially increasing the risk of hematological disorders and diminishing both innate and adaptive immunity.

Our findings indicate that *in-utero* PFOS exposure disrupted the metabolic milieu in the maternal and intrauterine environment, which was associated with perturbed transcriptional and metabolic profiles of FL-HSCs. PFOS-perturbed HSC developmental pathways at the FL microenvironment led to an expansion of HSC populations and specific activation of progenitors for granulocytes, megakaryocytes/erythrocytes, and lymphocytes. Our data reported for the first time that *in-utero* PFOS exposure during development can influence fetal hematopoiesis. Our findings justify further studies to examine whether the disruption of HSC developmental paths would have lasting effects on postnatal immune functions.

## Data Availability

The transcriptome and metabolomics data have been deposited with the BioProject accession number (PRJNA1227529) and the MetaboLights^110^ repository under the study identifier MTBLS12263, respectively..

## Supporting Information Available

Gating strategy in flow cytometry; PFOS concentrations and metabolome in AF and FL; cytokines in AL and FL; flow diagram of HSC enrichment, gene heatmap, and reactome enrichment; KEGG enrichment of FL-HSC; list of antibodies, maternal blood metabolome; AF metabolome; FL metabolome; HSC transcriptome; HSC metabolome; gating strategy in flow cytometry (Supplementary Figure S1); PFOS concentrations in amniotic fluid and fetal liver, metabolome, and the KEGG enrichment at gestational day 14.5 (Supplementary Figure S2); the levels of cytokines in amniotic fluid and fetal liver at gestational day 14.5 (Supplementary Figure S3); flow diagram demonstrating FL-HSC enrichment, gene clustering heatmap, and reactome enrichment analysis (Supplementary Figure S4); the KEGG enrichment of FL-HSC at gestational day 14.5 (Supplementary Figure S5) (PDF)
Antibodies for flow cytometry (Supplementary Table S1) (PDF)
The metabolome of maternal blood plasma at gestational day 14.5 (Supplementary Table S2) (XLSX)
The metabolome of amniotic fluids at gestational day 14.5 (Supplementary Table S3) (XLSX)
The metabolome of fetal livers at gestational day 14.5 (Supplementary Table S4) (XLSX)
The transcriptome of FL-HSCs at gestational day 14.5 (PRJNA 1227529) (Supplementary Table S5) (XLSX)
The metabolome of FL-HSCs at gestational day 14.5 (Supplementary Table S6) (XLSX)
