# Supplementary material for: Impact of PFOS Exposure on Murine Fetal Hematopoietic Stem Cells, Associated with Intrauterine Metabolic Perturbation
Source: Environ Sci Technol. 2025 Mar 13;59(11):5496–509. doi: 10.1021/acs.est.5c02623 (PMC11948485; doi:10.1021/acs.est.5c02623)
Supplement: Supplementary file 1 — es5c02623_si_001.pdf [file es5c02623_si_001.pdf]

Impact of PFOS Exposure on Murine Fetal Hematopoietic Stem Cells, Associated with Intrauterine Metabolic Perturbation.

Wang Ka LEE[1], Hin Ting WAN[1], Zheyu CHENG[1], Wing Yee CHAN[1], Thomas Ka Yam LAM[2], Keng Po LAI[3], Jianing WANG[2], Zongwei CAI[2], Chris Kong Chu WONG[1,2*]

[1]Croucher Institute for Environmental Sciences, Department of Biology, Hong Kong Baptist University, Hong Kong SAR; [2]State Key Laboratory in Environmental and Biological Analysis, Hong Kong Baptist University, [3] Departement of Applied Science, Hong Kong Metropolitan University, Hong Kong SAR.

Supporting Information

Summary: 14 pages, 5 figures, 6 tables.

Supplementary Fig S1. Gating strategy in flow cytometry
Supplementary Fig S2. PFOS concentrations in amniotic fluid and fetal liver, metabolome, and the KEGG enrichment at gestational day 14.5.
Supplementary Fig S3. The levels of cytokines in amniotic fluid and fetal liver at gestational day 14.5.
Supplementary Fig S4. Flow diagram demonstrating FL-HSC enrichment, gene clustering heatmap, and reactome enrichment analysis.
Supplementary Fig S5. The KEGG enrichment of FL-HSC at gestational day 14.5.

Supplementary Table S1. Antibodies for flow cytometry.
Supplementary Table S2. The metabolome of maternal blood plasma at gestational day 14.5.
Supplementary Table S3. The metabolome of amniotic fluids at gestational day 14.5.
Supplementary Table S4. The metabolome of fetal livers at gestational day 14.5.
Supplementary Table S5. Transcriptome of FL-HSCs at gestational day 14.5.
Supplementary Table S6. The metabolome of FL-HSCs at gestational day 14.5.

Suppl Fig S1

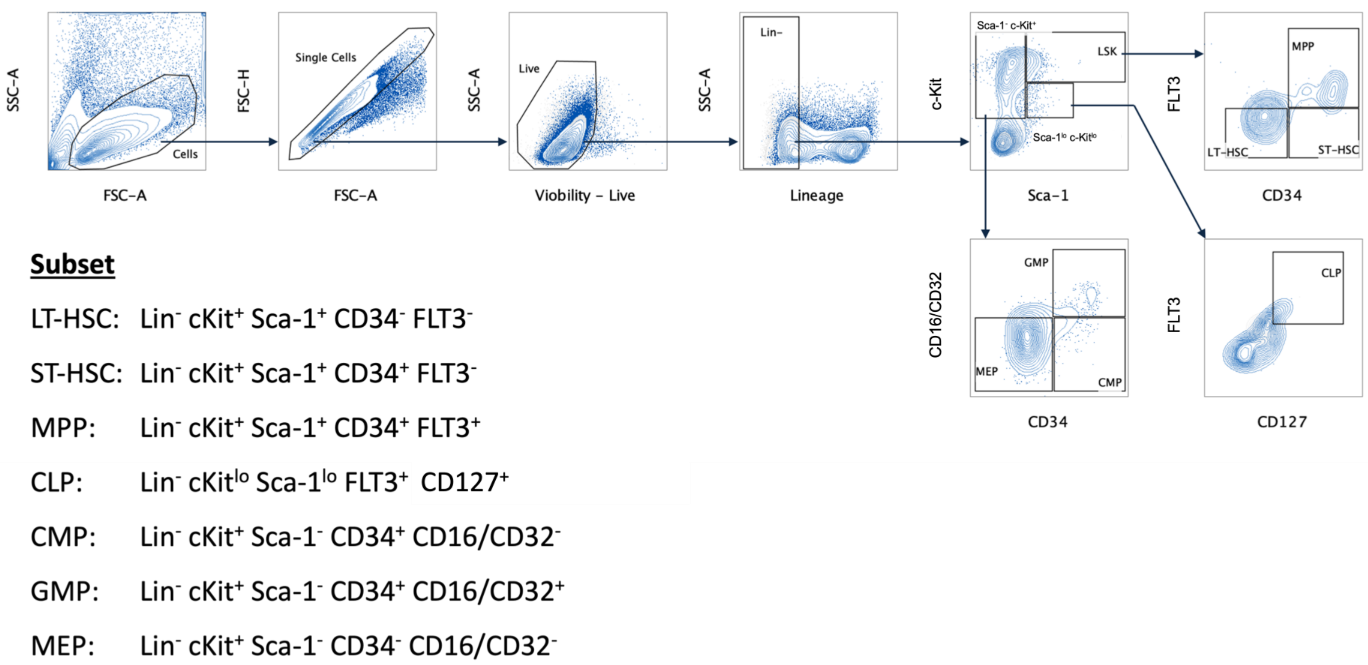

Suppl Fig S1. Gating strategy: The SSC-A and FSC-A channels separated the debris, followed by FSC-H and FSC-A channels to identify single cells. Viobility 405/452 fixable dye was used to identify the live cells. Firstly, lineage-negative (Lin$^-$) cells were gated, followed by c-Kit and Sca-1 channels to classify the cells into three groups: (i) Sca-1$^-$ c-Kit$^+$, (ii) Sca-1$^+$ c-Kit$^+$ (LSK), and (iii) Sca-1^{lo} c-Kit^{lo}. For (i) Sca-1$^-$ c-Kit$^+$, the cells were further classified with CD34 and CD16/CD32 markers into common myeloid progenitor (CMP: Lin$^-$, Sca-1$^-$, c-Kit$^+$, CD34$^+$, CD16/CD32$^-$), granulocyte-macrophages progenitors (GMP: Lin$^-$, Sca-1$^-$, c-Kit$^+$, CD34$^+$, CD16/CD32$^+$), and megakaryocyte-erythrocyte progenitor (MEP: Lin$^-$, Sca-1$^-$, c-Kit$^+$, CD34$^-$, CD16/CD32$^-$). For (ii) Sca-1$^+$ c-Kit$^+$, the cells were further classified with CD34 and Flt3 markers into long-term hematopoietic stem cell (LT-HSC: Lin$^-$, Sca1$^+$, cKit$^+$, CD34$^-$, Flt3$^-$), short-term HSC (ST-HSC: Lin$^-$, Sca1$^+$, cKit$^+$, CD34$^+$, Flt3$^-$), and multipotent progenitors (MPP: Lin$^-$, Sca1$^+$, cKit$^+$, CD34$^+$, Flt3$^+$). For (iii) Sca-1^{lo} c-Kit^{lo}, the cells were classified with Flt3 and CD127 markers into common lymphoid progenitor (CLP: Lin$^-$, Sca1^{lo}, cKit^{lo}, Flt3$^+$, CD127$^+$).

Suppl Fig S2

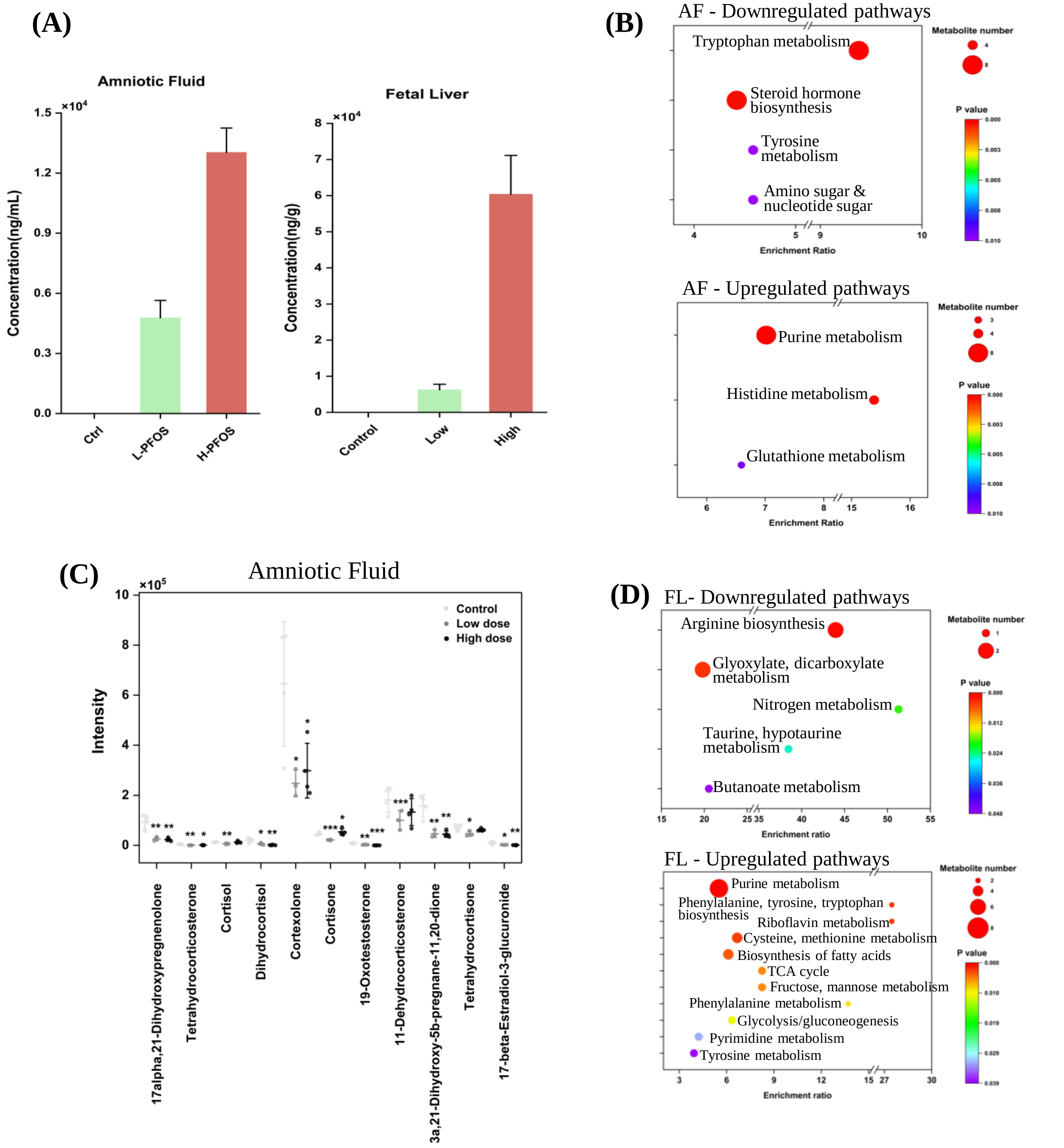

Suppl Fig S2. The metabolome of amniotic fluid (AF) and fetal liver (FL) at GD 14.5 (A) PFOS concentrations in AF and FL of the control and PFOS groups. **(B)** The KEGG enrichment of the AF metabolome. The abscissa in the graph is the ratio of the number of differential metabolites on the KEGG pathway to the total number of differential metabolites, and the ordinate is the KEGG pathway. The panels show the down- and up-regulated pathways in PFOS treatment versus the control. **(C)** The downregulated steroid hormone synthesis pathways in PFOS-exposed groups showed a significant reduction in dihydroxy-pregnenolone, glucocorticoids, and androgen concentrations in AF. **(D)** The KEGG enrichment of the FL metabolome.

Suppl Fig S3

(A)

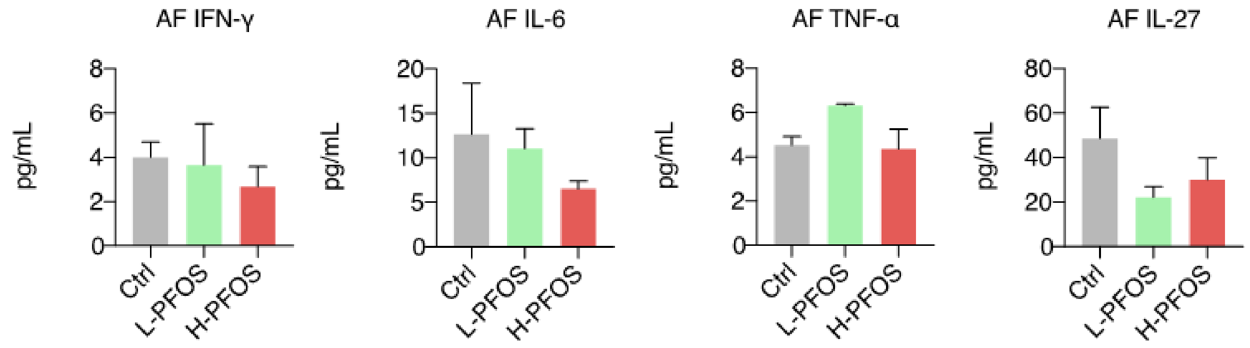

(B)

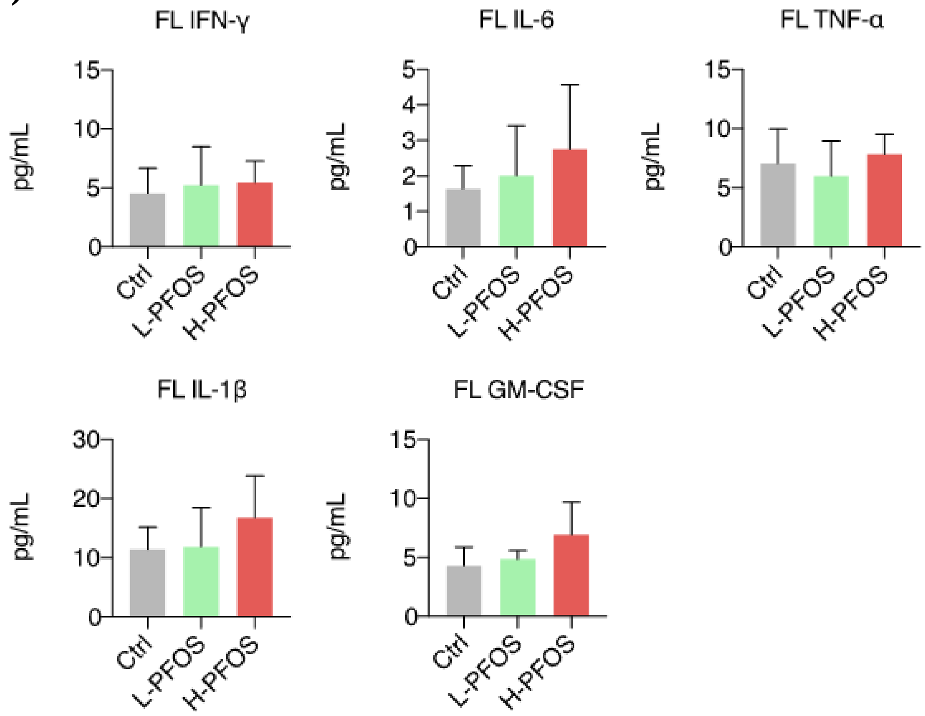

Suppl Fig S3. The levels of cytokines in amniotic fluid (AF) and fetal liver (FL) at gestational day 14.5. The IFN-γ, IL-6, and TNF-α levels showed no significant differences between the control and PFOS groups in **(A)** AF and **(B)** FL samples. IL-27 in AF and IL-1β & GM-CSF in FL showed no significant difference between the control and PFOS groups.

Suppl Fig S4

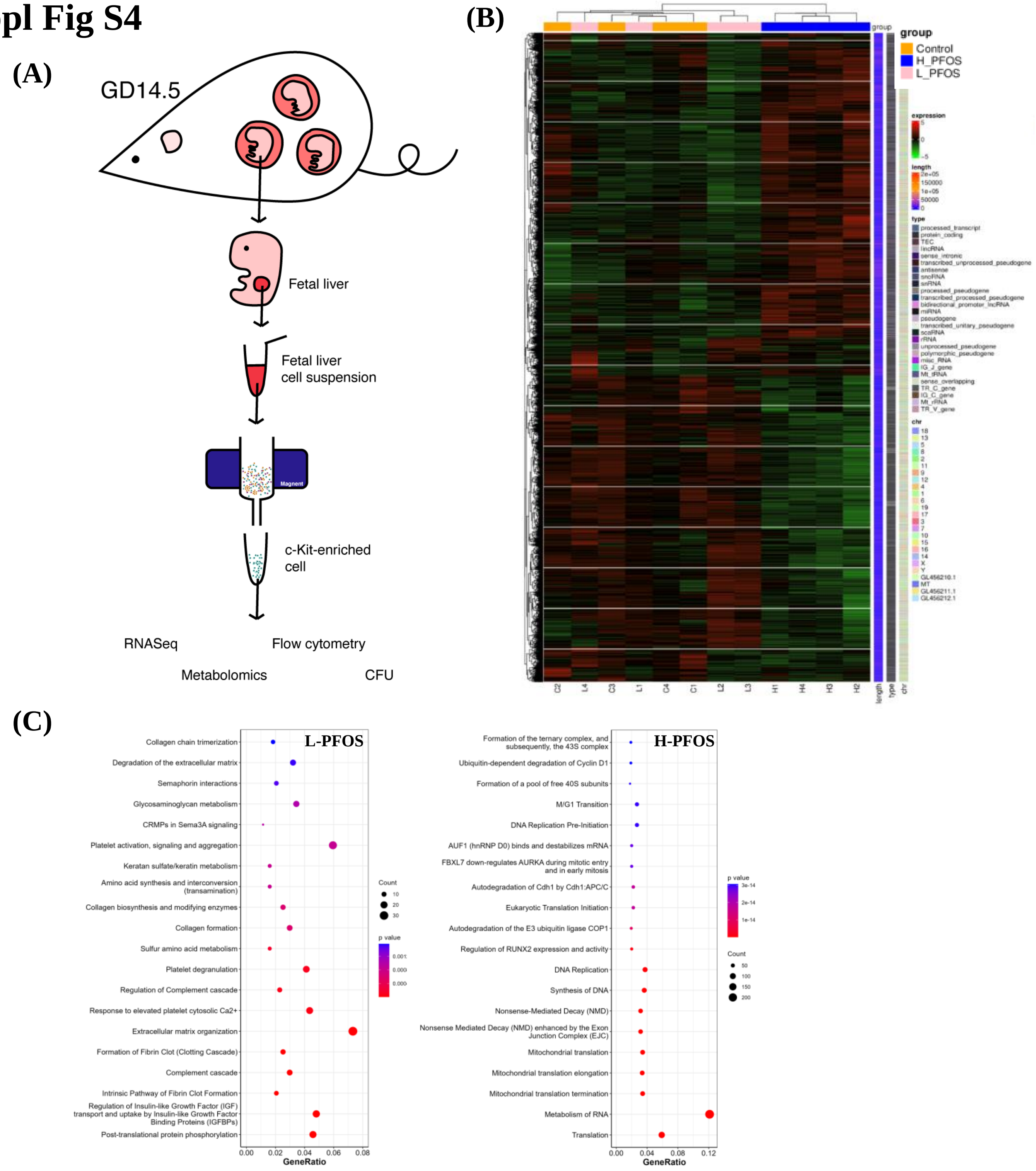

Suppl Fig S4. Fetal liver hematopoietic stem and progenitor cells (FL-HSCs) at GD 14.5. (A) A diagram shows the HSC-isolation approach. **(B) Transcriptome.** Differential expression gene clustering heatmap. The overall results of the FPKM cluster analysis were clustered using the log2(FPKM+1) value. In the visualization, red color represents genes with high expression levels, while blue color represents genes with low expression levels. The color gradient from red to blue indicates a range of log2(FPKM+1) values from large to small. **(C) Reactome enrichment analysis.** The x-axis on the graph represents the ratio of the number of differential genes to the total number of differential genes in the Reactome pathway. The y-axis represents the Reactome pathway. The graph displays the 20 most significant pathways. The size of a point on the graph indicates the number of genes associated with a specific pathway, and the color, ranging from red to purple, indicates the level of enrichment significance.

Suppl Fig S5

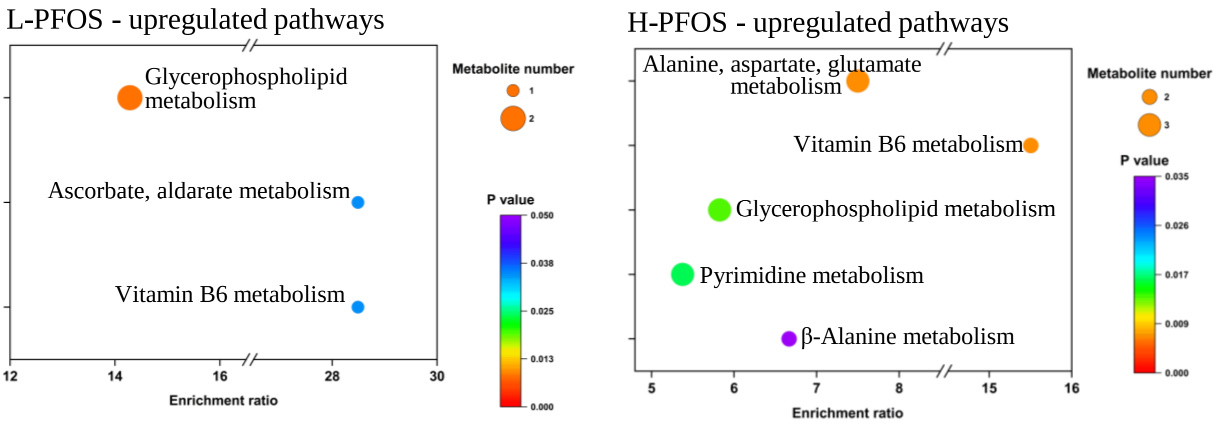

Suppl Fig S5. The metabolome of FL-hematopoietic stem cells (HSCs) at GD14.5. The KEGG enrichment of L-PFOS and H-PFOS groups. The abscissa in the graph is the ratio of the number of differential metabolites on the KEGG pathway to the total number of differential metabolites, and the ordinate is the KEGG pathway. The panels show the upregulated pathways in PFOS treatment versus the control.
