# Supplementary material for: Impact of PFOS Exposure on Murine Fetal Hematopoietic Stem Cells, Associated with Intrauterine Metabolic Perturbation
Source: Environ Sci Technol. 2025 Mar 13;59(11):5496–509. doi: 10.1021/acs.est.5c02623 (PMC11948485; doi:10.1021/acs.est.5c02623)
Supplement: Supplementary file 2 — es5c02623_si_002.pdf [file es5c02623_si_002.pdf]

Supplementary Table S1. Antibodies for flow cytometry.

Hematopoietic Lineage Labeling Cocktail, Biotin	Miltenyi Biotec (130-092-613)
Biotin Antibody, VioGreen	Miltenyi Biotec (130-113-297)
CD150 (SLAM) Antibody, PE	Miltenyi Biotec (130-125-987)
CD34 Antibody, FITC	Miltenyi Biotec (130-117-775)
CD127 Antibody, APC	Miltenyi Biotec (130-122-938)
Sca-1 Antibody, PerCP-Vio 700	Miltenyi Biotec (130-128-803)
CD117 Antibody, PE-Vio 770	Miltenyi Biotec (130-111-695)
CD16/32 Antibody, APC-Vio 770	Miltenyi Biotec (130-129-760)
CD135 Antibody, PE/Cyanine5	BioLegend (135312)
Viobility 405/452 Fixable Dye	Miltenyi Biotec (130-130-420)
